# Conference report: the importance of the gut microbiome and nutrition on health

**DOI:** 10.1017/gmb.2021.4

**Published:** 2021-12-20

**Authors:** Derek Ball, Spiridoula Athanasiadou

**Affiliations:** 1 Institute of Education in Healthcare and Medical Sciences, University of Aberdeen, Aberdeen, UK; 2 Scotland’s Rural College, Edinburgh, United Kingdom

**Keywords:** Gut, Microbiome, Nutrition, Health

## Abstract

The Nutrition Society Spring Conference (28–29 March 2021) focussed on the gut microbiome and health that was divided across three separate but inter-related areas from the impact of nutrition on the gut microbiome, the cause and effect of nutrition and health on the gut microbiome to the interaction between pathogens and gut microbiota. The program was supported by two plenary lectures, the first discussed the computational methods commonly employed to examine gut microbiota and the concluding lecture presented the interaction between gut microbiome, nutrition and health in older populations.

Analysing the complexity of the microbiome requires an interdisciplinary approach utilising molecular biology and bioinformatics. Dr Laura Glendinning (Roslin Institute, Edinburgh, UK) provided an excellent insight to the various computational approaches available to analysing the microbiota populations (Glendinning et al., 2020). She highlighted the requirements for good analytical practice based on; the type of data being generated; the experimental hypothesis being tested and the challenges arising to analyse the data. Further discussion focussed on the applications for analysing the taxonomy that exists in samples, that while informative do not necessarily indicate function. The discussion was expanded into the application of metagenomics to identify diversity changes and the identification of microbiota clustering with identification of the limitations to collecting and presenting data. A key message from the lecture was the importance of good communication between the wet and dry laboratories to both maximise and optimise data analysis.

The use of animal models to understand the link between the gut microbiome, nutrition and growth was presented by Dr Gillian Gardiner (Waterford Institute of Technology, Ireland). Using a porcine model, Dr Gardiner discussed the relationship between gut microbiota, immunomodulation, feed efficiency and growth. The identification of farm-specific biomarkers was highlighted as a strategy to understand the relationship between feed efficiency and growth as opposed to identify feed-specific microbiota due to the variability as a function of the rearing environments. Furthermore, Dr Gardiner illustrated that reprogramming either the maternal or foetal microbiome did not necessarily restore the feed efficiency phenotype, and in some cases led to a retardation in growth.

Linking the role of the gut microbiota on the availability and activity of a range of metabolites was presented by Prof Ian Rowland (University of Reading, UK). Microbiota metabolic activity significantly enhances the host’s capacity to metabolise a wide range of dietary components that subsequently extends the range of formed metabolites. Using the example of short-chain fatty acids, acetate, butyrate and propionate, Professor Rowland demonstrated that fermentation of dietary carbohydrates by the microbiota, that increases short-chain fatty acid availability, not only led to metabolite expansion, but could also act as cell-signalling molecules (Rowland et al., 2018). He concluded his presentation that the large inter-individual variation in gut microbiota has consequences on both metabolism and health.

The interplay between dietary fibre intake, gut microbiota and health was discussed by Prof Christine Edwards (University of Glasgow, UK). Focussing on the role of dietary fibre, Professor Edwards provided evidence of the importance of dietary fibre on gut microbiota as a source of energy and that these can be present as a complex matrix of non-digestible components. A range of factors that influence the interaction between microbiota and dietary fibre was discussed that included method of presentation, dosage, interaction between dietary fibres (Edwards et al., 2017). Understanding the complex nature of this relationship was further illustrated by reference to barriers to dietary fibre intake, differences in gut physiology between individuals and the limitations of current models and tracers and the reporting of these studies.

Manipulation of the microbiota to improve gut health in individuals with an underlying condition was addressed by Prof Konstatinos Gerasimidis (University of Glasgow, UK). Focussing on Crohn’s Disease (CD), Prof Gerasimidis provided evidence that enteral nutrition in paediatric patients could reduce the biomarkers of gut inflammation during an 8-week period by alteration of the gut microbiota. There are limitations to this approach in relation to the volume of enteral nutrition required (120 L/week) and that following the cessation of treatment the symptoms had returned. He explained that alternative approaches were now being trialled using a novel ordinary diet-based therapy that was proving effective in children with CD.

Returning to the theme of short-chain fatty acids (SCFA), Prof Gary Frost (King’s College, London, UK) and Dr Douglas Morrison (University of Glasgow, UK), explained that the gut microbiota generate short-chain fatty acids in the large intestine. In support of previous evidence, they discussed the multi-factorial effects that SCFA’s play in several tissues and highlighted the limited evidence from human studies that suggested a clear line for future study. Focussing on propionate they presented data from studies utilising inulin, as a vehicle to deliver propionate directly to the colon, and identified an effect from propionate on appetite regulation, beta cell function and glucose homeostasis as well as cardio-metabolic markers.

On day 2, the session started with a discussion on the effects of parasitic worm infection on the gut microbiome. Dr Lisa Reynolds (University of Victoria, Canada) provided a fascinating insight into Helminth infection on the small intestinal metabolome. Although parasitic worm infection can increase the risk of infection to the host, Dr Reynolds also explained that Helminth infection can also be immunosuppressive (Brosschot and Reynold, 2018) and for conditions where intestinal inflammation is a major pathology these treatment-induced infections can alleviate symptoms. Furthermore, the relationship between the local bacterial microbiota, metabolome and Helminth infection was illustrated by the changes in isovalerate levels.

The final two presentations of the session focussed on specific pathologies within the intestinal tract that encompassed inflammatory bowel disease (IBD), delivered by Prof Georgina Hold (University of New South Wales, Australia) and gastric cancer, covered by Dr Amanda Rossiter (University of Birmingham, UK). Prof Hold explained that while the rate of IBD appears to be low (0.3% prevalence), the rates continue to increase and that microbial composition as evidenced through 16S rRNA sequencing expression is highly linked to classifying disease status. She further highlighted that metagenomics appears to be the best method to identify treatment response. Furthermore, Prof Hold stressed that a holistic understanding of the intestinal microbiota is essential if we are to comprehend the composition, function and metabolic capacity that can affect disease treatment. Focussing on the upper gastrointestinal tract, Dr Rossiter discussed the link between the bacterium, *Helicobacter pylori*, and gastric cancer and the relationship with other bacterial species and concluded that these relationship are not fully understood. She then presented data on *H. pylori* growth in a polymicrobial environment with the observation that *Actinomyces oris* completely inhibits *H. pylori* growth. However, she also indicated that co-infection with *H. pylori* and *Actinomyces spp* led to an increase in interleukin 8 expression in a gastric cell carcinoma model that could possibly exacerbate the inflammatory response. She concluded that understanding the gastric microbiota is required if we asre to unlock the mechanism between the bacterial population and gastric carcinogenesis.

The second plenary lecture by Prof. Paul O’Toole (University College Cork, Ireland) discussed the diet-microbiome and health axis from the perspective of the ageing population. He described how the gut microbiome in an aged population differs significantly from a young population (O’Toole and Jeffery, 2018) and this could be due to several factors and that one of these is a reduction in diet diversity. The changes in dietary habits correlates with increasing frailty and independence of residence. Changes in dietary intake, either by the use of probiotics or the adoption of a Mediterranean diet results in measurable changes in microbiota profile in humans. In addition, he reported that there is a positive relationship between the adoption of a Mediterranean diet and a delay in the onset of ageing-related health loss. He also highlighted the differences in body mass index between individuals that reside in the community and those in residential care and the impact this would have on metabolic status and that non-dietary factors should not be overlooked.

In summary, the data presented and the roundtable discussions that took place after the presentation sessions illustrated the diversity of impacts from the gut microbiome on health, metabolic status, development and growth and maintenance through the lifespan. A significant number of presentations also discussed the merits and limitations of the different methodologies applied to understand the microbiota profile and the various models, from cell to organism and animal/human, to examine the function of the gut microbiota and its impact on whole body function.
